# Analysis of Elastic Properties of Polypropylene Composite Materials with Ultra-High Molecular Weight Polyethylene Spherical Reinforcement

**DOI:** 10.3390/ma15165602

**Published:** 2022-08-15

**Authors:** Jong-Hwan Yun, Yu-Jae Jeon, Min-Soo Kang

**Affiliations:** 1 Mobility Materials-Parts-Equipment Center, Kongju National University, Gongju-si 32588, Korea; 2 Department of Medical Rehabilitation Science, Yeoju Institute of Technology, Yeoju 12652, Korea; 3 Division of Smart Automotive Engineering, Sun Moon University, Asan-si 31460, Korea

**Keywords:** UHMWPE, polypropylene, elastic properties, finite element analysis

## Abstract

This study proposes an isotropic composite material with enhanced elastic properties based on a reinforcement mechanism using ultra-high molecular weight polyethylene (UHMWPE) spherical molecules. Elastic properties are predicted through finite element analysis by randomly mixing UHMWPE using polypropylene (PP) as a matrix. The change in elastic properties of the composite is calculated for volume fractions of UHMWPE from 10 to 70%. Furthermore, the results of finite element analysis are compared and analyzed using a numerical approach. The results show that the physical properties of the composite material are enhanced by the excellent elastic properties of the UHMWPE, and the finite element analysis results confirm that it is effective up to a volume fraction of 35%.

## 1. Introduction

A composite material can be defined as an assembly of two or more materials with properties superior to those of each of the constituent materials [1,2,3,4,5,6]. Composite materials formed by embedding a material in a matrix are commonly called reinforcement arrangements (or fillers) [7,8,9,10]. The matrix directly receives the cohesive force and direction of the load. Moreover, the load that the composite material receives may be transmitted as internal stress. A composite material formed in this manner is highly heterogeneous and may be isotropic or anisotropic depending on the material used [11,12,13]. The properties of the matrix and the charge, shape, proportion of the charge, quality of the interface, and production process used are all parameters that can affect the properties of the composite material [14,15,16,17]. In general, a polymer material is most frequently used as the matrix, and various materials such as metal, ceramic, or plastic may be used as the reinforcing material. Such composite materials may exhibit a continuous or discontinuous mechanism according to the shape of the reinforcing material used. A composite mainly formed using fiber as a reinforcing material is an example of a composite material distributed in a continuous phase [18,19,20,21,22]. Continuous phase composite materials are mainly formed by mixing functional fiber material in fiber form with polymer material and are classified as anisotropic due to the directionality of the fiber. To use such anisotropic materials, a fiber-reinforced composite material is configured in a stacked structure to prevent a defect against warpage [23,24]. In contrast, for a composite material with a discontinuous mechanism, the composite is generally manufactured using a spherical additive or an additive in the form of a particle. Additives in a spherical or particle form are freely dispersed in the matrix, and thus, an isotropic composite material can be manufactured [25,26,27,28,29]. Considering such an isotropic composite material has no directionality, it has a high potential to be widely used in the manufacturing field. In this study, we proposed a composite material using ultra-high molecular weight polyethylene (UHMWPE) spherical particles as the additive to construct such an isotropic composite material.

UHMWPE is attracting significant attention from researchers and industrial engineers owing to its excellent mechanical properties, low density, high chemical resistance and impact strength, low hygroscopicity, and high wear resistance [30,31,32,33]. UHMWPE fiber witnessed a surge of research activities right after its commercialization in the late 1970s for a range of applications, including (1) ballistic protection, (2) aerospace, automotive, and defense applications, and, (3) increasingly, medical devices [34,35,36]. A composite material manufactured by mixing UHMWPE, which has excellent characteristics as an additive, in a polypropylene (PP) matrix would be expected to possess excellent physical properties. In this study, to predict and analyze these properties, a numerical and finite element analysis approach was used to investigate a composite material obtained by mixing UHMWPE with a diameter of 10 μm with PP. In addition, to develop a composite material capable of injection molding, an isotropic composite material is proposed. To develop an isotropic composite material, UHMWPE of a spherical material was used. The physical properties according to the volume fraction (10–70%) of the additive were calculated using micromechanics models (Voigt, Reuss, and Halpin–Tsai) to predict the linear elastic properties of the composite material. Finally, finite element method (FEM) was used to compare and analyze the change in physical properties according to the addition of the UHMWPE spherical particles using the homogenization method.

## 2. Micromechanics Models

The Voigt model and Reuss model are the two simplest and most classical models used for estimating the elastic modulus of composite materials [37,38,39]. The rule of mixture predicts the elastic properties of composite materials according to the volume ratio of the constituent materials using Voigt and Reuss models [40,41,42]. The Voigt and Reuss models are often used to set the bound of the elastic modulus in a composite made of two materials. As the Voigt model is subject to a uniform strain, an upper bound of the elastic modulus can be set. In contrast, the Reuss model may be considered a lower bound because it is subject to uniform stress. As shown in Equation (1), the Voigt model is similar to composite materials connected in series and is developed assuming that the two materials are simply and linearly synthesized according to the volume fraction. *E_c_* is the elastic modulus of the composite material, *C*_0_ is the volume fraction of PP, *C*_1_ is the volume fraction of UHMWPE, *E*_0_ is the elastic modulus of PP, and *E*_1_ is the elastic modulus of UHMWPE. In this formula, *n* = 2 because the synthesized composite material is composed of binary elements.

(1) $$E_{c}=\overset{n}{\underset{r=0}{\sum }}C_{r}E_{r}\;\;\;\;\;\;\;\;\;$$

The Reuss constitutive model is used to model elastic plasticity and the total Lagrangian formula is used to model the finite strain. In the Reuss model, the stiffness of the material is generally measured by the elastic modulus *E*; in the macroscopic elastic range, stiffness is the force required to cause a unit displacement. Its reciprocal 1/*E* is called compliance, which is the displacement caused by unit force. The Reuss model is presented in Equation (2).

(2) $$E_{c}={\left(\overset{n}{\underset{r=0}{\sum }}C_{r}\frac{1}{E_{r}}\right)}^{-1}\;\;$$

The Halpin-Tsai model is an improvement of the existing mixing law using reinforcement factors calculated through experiments. The reinforcing factor depends on the shape and arrangement of the additives and the loading conditions. The Halpin–Tsai model [43,44] is applied here to predict the compressive Young’s and shear moduli of the composite, which are dependent on the particle volume fraction (PVF). This Halpin–Tsai approach is simple and easy to use in the design process, and the semi-empirical equation can be expressed as Equation (3). *ξ* is a measure of particle filler that depends on particle geometry. In general, *ξ* follows Equation (4). Therefore, it has a value of 2 for spherical particles.

(3) $$E_{c}=\frac{E_{0}\cdot \left[E_{1}+\xi \cdot \left(C_{0}\cdot E_{0}+C_{1}\cdot E_{1}\right)\right]}{C_{0}\cdot E_{1}+C_{1}\cdot E_{0}+\xi \cdot E_{r}}\;\;$$

(4) $$\xi =\frac{2l}{d}\;\;\;$$

According to this numerical method, changes in physical properties of the reinforcing material UHMWPE in a PP matrix according to the volume fraction, from 10% to 70% UHMWPE, were calculated.

## 3. Homogenization Method

**Figure 1:**
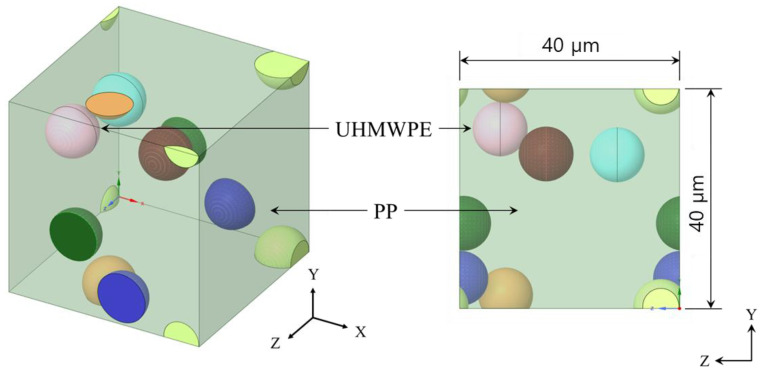
Ultra-high molecular weight polyethylene (UHMWPE) particle-reinforced polypropylene (PP) composite representative volume element modeling.

**Figure 2:**
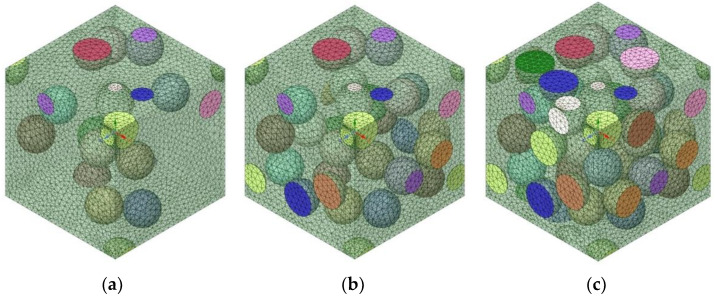
Mesh modeling with different ultra-high molecular weight polyethylene (UHMWPE) volume fractions: (**a**) 10%, (**b**) 20%, and (**c**) 30%.

In numerical homogenization, it is assumed that the representative volume element (RVE) or unit cell is locally repeated with a very small microstructure compared with the overall macroscopic dimensions of the structure of interest, where the different base materials are fully bonded in the RVE [45]. Computational homogenization methods and their inverse forms with the FEM have been considered to be rather effective for a range of problems [46,47,48]. Generation of the RVE plays an important role in determining the effective properties of composite materials using finite element techniques using the homogenization method [49]. To implement the finite element analysis modeling required for the homogenization method, UHMWPE was applied to the spherical reinforcing material and assumed to be a sphere with a diameter of 10 μm. The 10 μm UHMWPE powder was studied using PM-200 from MIPELON^TM^. To compare with the experimental results later, a finite element analysis was performed using 10 μm powder. The matrix material was PP, and the RVE was modeled to a size of 40 μm × 40 μm × 40 μm in consideration of the size of the reinforcement (Figure 1). In addition, for UHMWPE, an RVE model with irregular culture was used to reflect the characteristics of particles in the hexagonal matrix material. Finite element analysis was conducted to calculate the change in physical properties according to the volume fraction of UHMWPE from 10% to 70% in the PP matrix (Figure 2). The physical properties of PP and UHMWPE used in this study are tabulated in Table 1.

## 4. Results and Discussion

**Figure 3:**
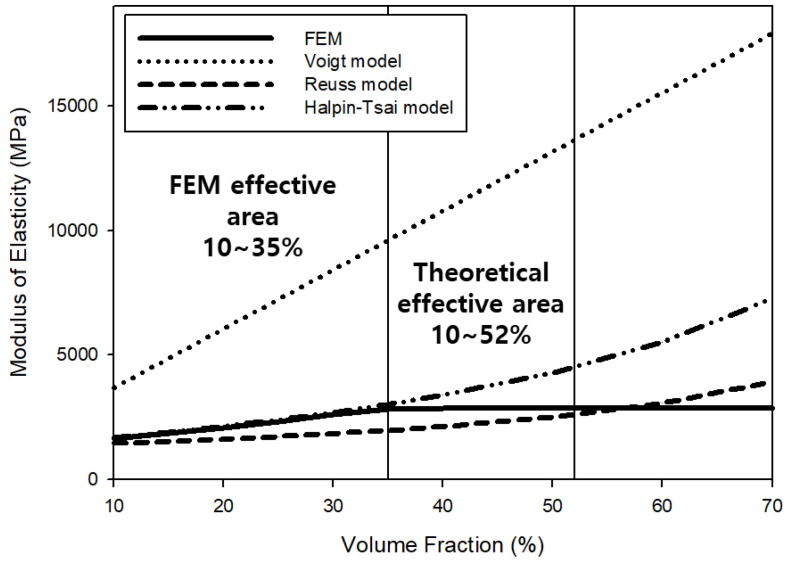
Values of Elastic modulus according to the volume fraction predicted by various models: Voigt, Reuss, Halpin–Tsai, and the finite element method (FEM).

**Figure 4:**
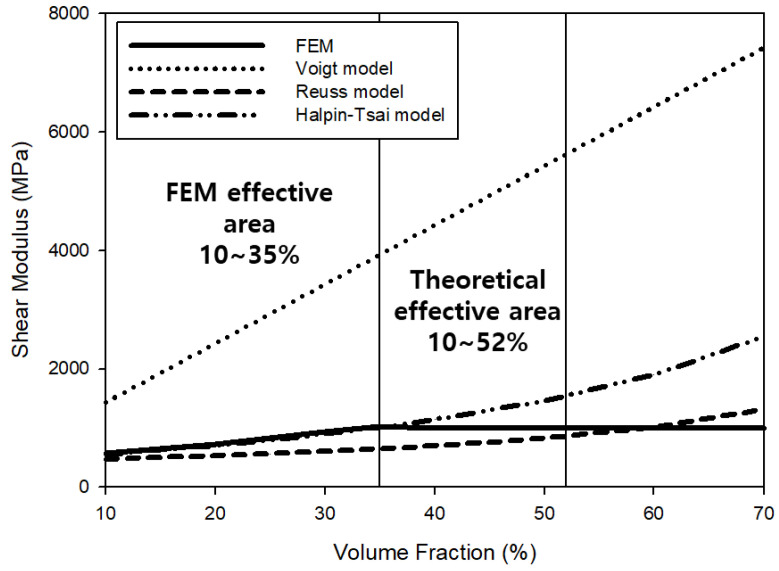
Values of shear modulus according to the volume fraction predicted by various models: Voigt, Reuss, Halpin–Tsai, and the finite element method (FEM).

Table 2 shows the numerical values according to the micromechanics model. In addition, Figure 3 shows changes in the predicted elastic modulus according to the volume fraction. Based on the results of linearly calculating the elastic modulus using the Voigt model, the UHMWPE volume fraction of 10% was calculated to have a value of at most 1798.5 MPa. As a result of analyzing the elastic modulus using the Reuss model, the lowest value was 1350.6 MPa. The elastic modulus calculated using the Halpin–Tsai model was 1394.3 MPa, most similar to the average elastic modulus obtained through the FEM (1394.07 MPa). As the volume fraction linearly increased, the Voigt model elastic modulus linearly increased, and those calculated by the Reuss model and the Halpin–Tsai model also showed a tendency to increase because of the increased influence of the elastic modulus of UHMWPE with an increase in the volume fraction. As shown in Figure 4, similar results were obtained for the predicted shear modulus. Elastic properties, such as elastic modulus and shear modulus, increased as the volume fraction of UHMWPE increased due to the excellent physical properties of UHMWPE (Figure 3 and Figure 4). In general, numerically accessible micromchanics models rely on volume fractions to yield elastic properties. Therefore, theoretically, as the volume fraction of UHMWPE increases, physical properties may be improved [50,51]. Furthermore, the results of predicting the elastic properties of the composite material using the FEM showed a tendency most similar to the elastic modulus and the shear modulus values calculated using the Halpin–Tsai model.

**Figure 5:**
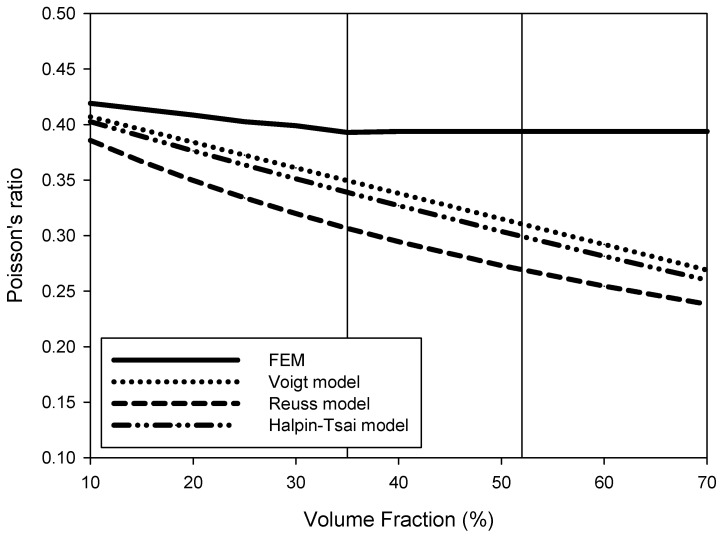
Values of Poisson’s ratio according to the volume fraction predicted by various models: Voigt, Reuss, Halpin–Tsai, and the finite element method (FEM).

As shown in Figure 5, the Poisson’s ratio of the PP-UHMWPE composite material calculated by numerical and FEM analysis varied with the volume fraction of composite material formation according to the difference in Poisson’s ratio between PP and UHMWPE. The Halpin–Tsai model and the FEM model showed the most similar tendencies in the prediction model for the elastic modulus and the shear modulus, but the FEM did not match any numerical analysis model in the calculation of the Poisson’s ratio.

**Figure 6:**
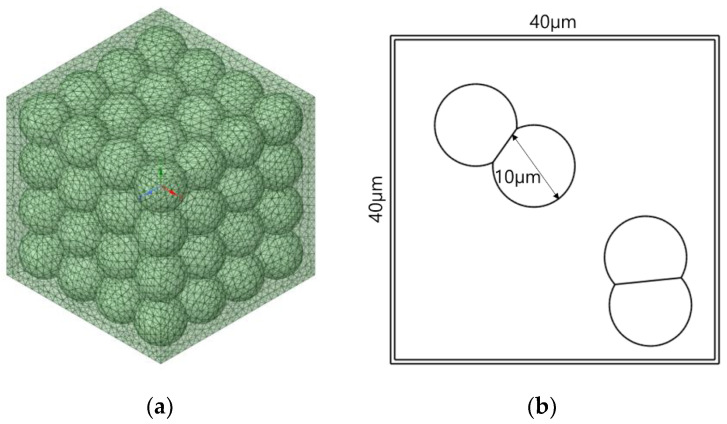
Spherical ultra-high molecular weight polyethylene (UHMWPE) particles at a volume fraction of 35% or more: (**a**) regular arrangement of spherical UHMWPE and (**b**) overlapping influence of spherical UHMWPE.

The size of the RVE used for finite element analysis in this study was 40 × 40 × 40 μm, and the spherical size of UHMWPE was 10 μm. Therefore, for regularly arranged UHMWPE spheres, up to 64 spheres can theoretically be arranged as presented in Figure 6a, and accordingly, the maximum volume fraction can be expected to be 52%. However, based on the results of calculating the physical properties through the FEM, it was confirmed that the volume fraction of UHMWPE was constant after 35%, as shown in Figure 3, Figure 4 and Figure 5 because it represents the maximum at a volume fraction of 35%, depending on the batch model of the UHMWPE reinforcing material randomly formed into the RVE lattice. According to the FEM analysis results, when the volume fraction of UHMWPE was 35% or more in the RVE, the spherical UHMWPE additive had directionality caused by overlapping the inner spherical UHMWPE powder as shown in Figure 6b. Therefore, it can be concluded that physical property analysis according to the influence of the tensor should be performed with anisotropic material above 35% volume fraction of UHMWPE. To this end, the elastic modulus and shear modulus of the PP-UHMWPE composite material were found to be similar in the Halpin–Tsai model and the FEM model up to 35% volume fraction of UHMWPE, but the results obtained from the FEM analysis are not valid above 35% volume fraction. Furthermore, it can be determined that the UHMWPE spherical composite material of 10 μm can be applied up to the theoretically maximum volume fraction of UHMWPE of 52%. UHMWPE powder of 10 μm or less may be used to ensure a higher volume fraction of UHMWPE and thereby enhance physical and elastic properties.

## 5. Conclusions

In this study, physical properties were predicted using numerical analysis and finite element analysis to predict the elastic properties of composite materials using 10 μm UHMWPE spherical powder as a reinforcing material and PP as a matrix. As spherical powder was used as the reinforcing material, the PP-UHMWPE powder composite could be modeled as an isotropic material. The elastic properties according to the volume fraction of the UHMWPE reinforcing material were compared and analyzed. As numerical methods, the Voigt, Reuss, and Halpin–Tsai models were used, and for the finite element analysis, the homogenization method using RVE identification was used for comparative analysis. The results are as follows.

(1) The linear calculation according to the volume fraction using the Voigt model was found to be the upper bound of the predicted elastic properties and showed a large error range when compared with the FEM analysis. In the calculation using the Reuss model, the prediction result of elastic properties was lower than that of the FEM analysis. The results of the calculation of elastic properties using the Halpin–Tsai model were found to be most similar to the FEM analysis.
(2) The powders of the spherical UHMWPE could theoretically be dispersed inside PP at a volume fraction of up to 52% if arranged in a lattice structure. However, in the FEM model, spherical powders of UHMWPE could be dispersed up to 35% volume fraction when they were randomly arranged.
(3) As a result of comparing and analyzing the numerical finite element analysis results for predicting the elastic properties of PP-UHMWPE isotropic composite materials, it was confirmed that the results of finite element analysis are reliable up to 35% UHMWPE volume fraction and theoretically up to 52%.
(4) To improve elastic properties, UHMWPE powder of 10 μm or less should be used to form an isotropic composite material by increasing the volume fraction of UHMWPE.

In the future, it is necessary to conduct research to predict the elastic properties of anisotropic composite materials in the high-UHMWPE volume fraction state (55% or more), and there is also a need to study changes in the physical orientation and elastic properties of the composite material according to the overlapping of the spherical UHMWPE powders.

## Figures and Tables

**Table 1:** Properties of polypropylene (PP) and ultra-high molecular weight polyethylene (UHMWPE).

No.	Properties	PP	UHMWPE
1	Elastic modulus (MPa)	1325	25,000
2	Shear modulus (MPa)	432.29	10,417
3	Poisson’s ratio	0.43	0.20
4	Bulk modulus (MPa)	3154.8	13,889.0
5	Density (kg/m^3^)	904	950

**Table 2:** Tubular data of micromechanics.

		2%	4%	6%	8%	10%	15%	20%	25%	30%	35%	40%	50%	60%	70%
Voigt model	E	1798.5	2272.0	2745.5	3219.0	3692.5	4876.3	6060.0	7243.8	8427.5	9611.3	10,795.0	13,162.5	15,530.0	17,897.5
	G	632.0	831.7	1031.4	1231.1	1430.8	1930.0	2429.2	2928.5	3427.7	3926.9	4426.2	5424.6	6423.1	7421.6
	nu	0.43	0.42	0.42	0.41	0.41	0.40	0.38	0.37	0.36	0.35	0.34	0.32	0.29	0.27
Reuss model	E	1350.6	1377.2	1404.8	1433.6	1463.6	1544.4	1634.6	1736.0	1850.8	1981.9	2133.0	2516.6	3068.6	3930.6
	G	440.7	449.5	458.7	468.2	478.1	504.9	534.8	568.5	606.8	650.5	701.1	830.1	1017.4	1313.8
	nu	0.42	0.41	0.40	0.39	0.39	0.37	0.35	0.33	0.32	0.31	0.29	0.27	0.25	0.24
Halpin-Tsai	E	1394.3	1466.0	1540.3	1617.3	1697.2	1910.8	2146.4	2407.6	2699.0	3026.0	3395.6	4300.8	5524.7	7271.8
	G	455.7	479.9	505.0	531.1	558.2	630.8	711.2	800.8	901.1	1014.3	1143.0	1461.7	1900.8	2544.0
	nu	0.42	0.42	0.41	0.41	0.40	0.39	0.38	0.36	0.35	0.34	0.33	0.30	0.28	0.26
